# Gene-specific long-term course, neurodevelopmental outcome and quality of life in patients with *LIS1/PAFAH1B1-, DCX-, DYNC1H1-, TUBA1A-* and *TUBG1*-related lissencephaly

**DOI:** 10.1186/s13023-026-04398-z

**Published:** 2026-05-23

**Authors:** Christiane R. Proepper, Lisa-Maria Schwarz, Sofia M. Schuetz, Katja von Au, Thomas Bast, Nathalie Beaud, Ingo Borggraefe, Friedrich Bosch, Melanie Busse, Jena Chung, Otfried Debus, Katharina Diepold, Thomas Fries, Gero von Gersdorff, Martin Haeussler, Andreas Hahn, Till Hartlieb, Ralf Heiming, Peter Herkenrath, Gerhard Kluger, Jonas H. Kreth, Gerhard Kurlemann, Peter Moeller, Deborah J. Morris-Rosendahl, Axel Panzer, Heike Philippi, Sophia Ruegner, Carolina Toepfer, Silvia Vieker, Adelheid Wiemer-Kruel, Anika Winter, Gerhard Schuierer, Ute Hehr, Tobias Geis

**Affiliations:** 1 University Children’s Hospital Regensburg (KUNO), University Hospital Regensburg, Franz-Josef- Strauß-Allee 11, 93053 Regensburg, Germany; 2 Department of Neurology, Krankenhaus Barmherzige Brüder Regensburg, Prüfeninger Str. 86, 93049 Regensburg, Germany; 3 Department of Ophthalmology, University Hospital Regensburg, Franz-Josef-Strauß-Allee 11, 93053 Regensburg, Germany; 4 Department of Pediatrics, Vivantes Klinikum im Friedrichshain, Landsberger Allee 49, 10249 Berlin, Germany; 5 Epilepsy Center Kork, Landstr. 1, 77694 Kehl, Germany; 6 Pediatric Practice, Osterstr. 18, 25836 Garding, Germany; 7 Division of Pediatric Neurology, Developmental Medicine and Social Pediatrics, Department of Pediatrics, Dr. von Hauner Children´s Hospital, University Hospital, LMU Munich, Lindwurmstr. 4, 80337 Munich, Germany; 8 Department of Neuropediatrics, Children’s Hospital Fürth, Jakob-Henle-Str. 1, 90766 Fürth, Germany; 9 Social Pediatric Center, Evangelisches Krankenhaus Mülheim/Ruhr, Schulstr. 10 a, 45468 Mülheim an der Ruhr, Germany; 10 Department of Pediatrics and Adolescent Medicine, Johannes Kepler University Linz, Krankenhausstr. 26-30, Linz, 4020 Austria; 11 Department of Pediatrics, Clemenshospital, Düesbergweg 124, 48153 Münster, Germany; 12 Pediatric Practice, Albert-Schweitzer-Weg 7, 37154 Northeim, Germany; 13 Department of General Pediatrics, Center of Social Pediatrics, Asklepios Clinic Sankt Augustin, Arnold-Janssen-Str. 29, 53757 St. Augustin, Germany; 14 Department of Medicine II, Division of Nephrology, Faculty of Medicine and University Hospital Cologne, Kerpener Str. 62, 50937 Cologne, Germany; 15 Pediatric Neurology and Social Pediatrics, University Children´s Hospital, Josef-Schneider-Str. 2, 97084 Wuerzburg, Germany; 16 Department of Child Neurology, Justus- Liebig-University Gießen, Feulgenstr. 10-12, 35392 Gießen, Germany; 17 Center for Pediatric Neurology, Neurorehabilitation and Epileptology, Schoen-Clinic, Krankenhausstr. 20, 83569 Vogtareuth, Germany; 18 Research Programme for Rehabilitation, Transition and Palliation, Paracelsus Medical University Salzburg, Strubergasse 21, Salzburg, 5020 Austria; 19 Pediatric Practice, Marktstr. 11, 30890 Barsinghausen, Germany; 20 Department of Pediatrics, Faculty of Medicine, University Hospital Cologne, Kerpener Str. 62, 50937 Cologne, Germany; 21 Department of Pediatrics, Institute of Rehabilitation, Transition and Palliation of Neurologically ill Children, Paracelsus Medical University, Strubergasse 21, Salzburg, 5020 Austria; 22 ERN Epicare, Center for Pediatric Neurology, Neurorehabilitation and Epileptology, Schoen-Clinic Vogtareuth, Krankenhausstr. 20, 83569 Vogtareuth, Germany; 23 Social Pediatric Center Oberberg, Klinikum Oberberg, Breidenbrucher Str. 2, 51674 Wiehl, Germany; 24 Department of Pediatric Neurology, Bonifatius Hospital Lingen, Wilhelmstr. 13, 49808 Lingen (Ems), Germany; 25 Center for Developmental Diagnostics and Social Pediatrics, Klinikum Wolfsburg, Sauerbruchstr. 7, 38440 Wolfsburg, Germany; 26 Genomic Medicine, National Heart and Lung Institute, Imperial College London; Royal Brompton Hospital, Guy’s and St. Thomas’ NHS Foundation Trust, London, SW6 3NP UK; 27 Department of Neuropediatrics, Center for Epilepsy, DRK Westend Clinic Berlin, Spandauer Damm 130, 14050 Berlin, Germany; 28 Social Pediatric Center Frankfurt-Mitte, Theobald-Christ-Str. 16, 60316 Frankfurt am Main, Germany; 29 Pediatric Practice, Marktstr. 6, 74592 Kirchberg/Jagst, Germany; 30 Pediatric Practice, Untermarkt 45, 82418 Murnau, Germany; 31 Epileptology Focus Practice, Droote 48/50, 44328 Dortmund, Germany; 32 Department of Neuropediatrics, Jena University Hospital, Am Klinikum 1, 07747 Jena, Germany; 33 Center for Neuroradiology, University Clinics and Bezirksklinikum Regensburg, Universitätsstr. 84, 93053 Regensburg, Germany; 34 Center for Human Genetics, Luitpoldstr. 4, 93047 Regensburg, Germany; 35 University Children’s Hospital Regensburg (KUNO), Hospital St. Hedwig of the Order of St. John, University of Regensburg, Steinmetzstr. 1-3, 93049 Regensburg, Germany

**Keywords:** *LIS1/PAFAH1B1*, *DCX*, *DYNC1H1*, *TUBA1A*, *TUBG1*, Lissencephaly, Quality of Life, Neurodevelopmental outcome, Supportive therapies, Epidemiology

## Abstract

**Background:**

Classic lissencephaly is a malformation of cortical development that includes agyria and pachygyria. The major clinical symptoms are developmental impairment, muscular hypotonia, and drug-resistant epilepsy. The severity of the clinical phenotype depends on the associated gene and mutation. This study aimed to systematically investigate the genotype-specific course of the disease including neurodevelopmental outcome, medical complications, use of non-pharmacological supportive therapies, and its impact on the quality of life of the affected families.

**Methods:**

47 patients with genetically and radiologically confirmed lissencephaly were included with mutation in *LIS1/PAFAH1B1* (*n* = 38), *DCX* (*n* = 5 males), *DYNC1H1* (*n* = 2), *TUBA1A* (*n* = 1) and *TUBG1* (*n* = 1) genes. Standardized questionnaires were completed by families and treating pediatricians. Quality of life was assessed with the PedsQL™ Family Impact Module.

**Results:**

Prenatal abnormalities, most commonly microcephaly, were observed in 14/37 (38%) of *LIS1/PAFAH1B1* patients and 2/5 (40%) of *DCX* patients. Early symptoms included microcephaly, developmental delay, muscular hypotonia, and epileptic seizures. The median age at suspected diagnosis was 5 months for *LIS1/PAFAH1B1* patients and 9 months for *DCX* patients. Compared to *LIS1/PAFAH1B1*, *DCX*-related lissencephaly patients showed significantly better neurodevelopmental outcome in reaching more advanced milestones such as walking unassisted (*z*=-2.23, *p* = 0.026) and speaking sentences (*z*=-2.53, *p* = 0.011). Frequent medical complications included recurrent respiratory infections (14/38 (37%) of *LIS1/PAFAH1B1* patients; 1/4 (25%) of *DCX* patients) and dysphagia/ vomiting (23/37 (62%); 2/4 (50%)), which may require tube feeding (15/38 (40%); 1/5 (20%)). A median of eight different supportive therapies was used per patient (range 1–17), with physiotherapy and respiratory therapy considered the most effective. The scores obtained for health-related quality of life (HRQL) were low (parental HRQL mean 61.23; SD 16.79).

**Conclusions:**

Our study confirms the severely impaired developmental potential and frequent neurological and medical complications in lissencephaly patients from an early age. The psychomotor prognosis in *LIS1/PAFAH1B1*-related lissencephaly is significantly worse compared to *DCX*-related lissencephaly. Supportive therapies are used intensively and are considered to be very effective. The disease puts a high burden on caregivers and the entire family. This emphasizes the need for appropriate epilepsy treatment, personalized care for patients and professional support for their families.

**Supplementary Information:**

The online version contains supplementary material available at 10.1186/s13023-026-04398-z.

## Background

Classic lissencephaly – commonly known as “smooth brain” and herein referred to as lissencephaly – is a spectrum of rare malformations of the entire cerebral cortex, affecting only 11.7 to 40 per million births [1–3]. It is caused by a disruption of neuronal migration during embryonal cortex formation resulting in an enlarged (pachygyria) or absent gyration (agyria) and thickening of the cortex, which can vary in location and severity [4–6]. As early as in the second trimester, anomalies such as ventriculomegaly and delayed opercular development can be detected via ultrasound and serve as “red flags”, leading to early diagnosis [7, 8].

The first identified causative mutation was a heterozygous microdeletion within 17p13.3 in patients with Miller Dieker Syndrome, discovered in 1983 by Dobyns et al. [9]. This observation was followed by the identification of the *LIS1/PAFAH1B1* gene [10, 11]. Subsequently for many years, the *DCX* gene (also known as *XLIS*) remained then the only additional target for genetic evaluation of lissencephaly patients [12–14]. Causative mutations in the *LIS1/PAFAH1B1* and *DCX* genes still are by far the most common lissencephaly-associated genes, now followed by mutations in *DYNC1H1* and *TUBA1A* [15, 16]. Thanks to more advanced technologies in recent years like next generation sequencing and whole exome analysis, currently 31 causative genes have been described in the literature [3]. In parallel with advances in genetic diagnostics, a novel classification system for MR imaging findings has been developed, which has helped to simplify and concretize the previous classification system. This classification also supports the diagnostic approach in genetic testing [17, 18].

Patients with lissencephaly usually display a severe clinical phenotype with onset of symptoms in early infancy. Early-onset epilepsy may be the first symptom and major concern, commonly progressing to drug-resistant epilepsy at all ages. Infantile epileptic spasm syndrome (IESS) is a frequent first seizure type and has been found in 57% of lissencephaly patients [19]. Over the course of the disease, epilepsy progresses to various epileptic seizures types and other epileptic syndromes such as Lennox-Gastaut syndrome [1, 3, 19–22]. In addition, most patients with lissencephaly have profound developmental delay and severe muscular hypotonia. Advanced motor milestones such as independent walking are rarely achieved [23]. Survival of these patients is limited, with a reported mortality rate of approximately 50% by the age of 10 years [21, 23]. However, the clinical phenotype and severity of the disease are known to depend on the mutated gene [18]. In general, *DCX*-associated lissencephaly may follow a milder disease course compared to *LIS1/PAFAH1B1*-associated lissencephaly, which is often associated with the most severe end of the spectrum [12, 21, 24, 25]. Furthermore, a genotype-phenotype correlation can also be observed regarding cortex formation, with gene-specific changes in the resulting cortical architecture [26].

Only limited clinical data on the gene-specific long-term clinical course, neurodevelopmental outcome and specific medical complications have been published for this rare cortical malformation, and comparisons between different genetic cohorts are scarce. Our study aimed to systematically analyze the genotype-specific disease course including neurodevelopmental outcome, neurological and medical problems in five genetic subgroups with radiologically and genetically confirmed lissencephaly. In addition, novel results on the use of non-pharmacological supportive therapies and on the family impact of the disease are provided.

## Patients and methods

### Patients

A total of 47 patients diagnosed with lissencephaly were included in our study cohort aged from 0.3 to 25 years. 26 patients were recruited via the database of the Center for Human Genetics in Regensburg. Further recruitment was conducted through cooperation with the Germany-based lissencephaly self-support group Liss e.V. (*n* = 17) and a network of pediatric neurologists in Germany, Austria and Switzerland (ESNEK = Erhebungen seltener neurologischer Erkrankungen im Kindesalter) (*n* = 4). This cohort and the data on epilepsy and response to antiseizure medications have already been reported in a previous publication [19].

Inclusion criteria applied to our cohort were (I) radiological diagnosis of lissencephaly on cerebral MRI, (II) genetic confirmation with presence of a causal pathogenic variant in a gene related with lissencephaly, and (III) written informed consent from the legal guardians of the participants.

### Questionnaire assessment

Standardized questionnaires evaluating the clinical data and neurodevelopmental outcome as well as the effectiveness of treatments were answered by the patients’ families and the treating pediatricians (see supplementary material for the sample questionnaires). A smaller subgroup of families (*n* = 14) in addition provided data for a follow-up evaluation with a second questionnaire. For these 14 patients the more recent updated data were taken into account.

The neurologic assessment was performed by the treating pediatric neurologist or pediatrician. Neurodevelopmental outcome was evaluated in parent-reported questionnaires using an ordinal ranking scale (categories: always/ frequent/ rarely/ never achieved). The achievement of a developmental milestone was determined when “always” or “frequent” was described. If updated data were available with divergent results, the best psychomotor answer was taken into account. At the time of the assessment, 7 children were younger than 2 years old. Since some of the assessed milestones are physiologically not yet reached in these younger children, they were (partially) excluded from the subsequent analysis: 2 patients with mutation in the *LIS1/PAFAH1B1* gene who were younger than 1 year were excluded from the whole analysis, whereas 5 further patients between 1 and 2 years (4 with mutation in *LIS1/PAFAH1B1* and 1 with mutation in *TUBA1A*) were excluded from selected more complex developmental milestones (free standing, walking with support, free walking, speaking of single words, speaking of whole sentences, pointing at persons/ objects, appointing objects on pictures).

The effectiveness of supportive therapies was rated by the parents on a subjective basis with ordinal categories (good/ partial/ no effect or clinical worsening).

In addition, we assessed health-related quality of life (HRQL) and family functioning using the standardized PedsQL™ Family Impact Module questionnaire. This validated module was established to measure the impact of pediatric chronic health conditions on parents and the family [27]. A validated German version of the questionnaire is available and was used for this study [28]. The questionnaire consists of 36 items covering 8 dimensions (physical functioning, emotional functioning, social functioning, cognitive functioning, communication, worry, daily activities and family relationships). A five-point scale is used to record the responses (0 = never a problem; 4 = always a problem). The items are reversed and linearly transformed into a 0-100 scale (0 = 100, 1 = 75, 2 = 50, 3 = 25, 4 = 0), with lower values indicating a higher HRQL burden for the caregivers. Of the 41 families contacted, valid HRQL questionnaires for evaluation were available for 18 patients. None of the HRQoL questionnaires received were considered invalid.

### Genetic and radiologic testing

All patients were genetically tested prior to inclusion in our study cohort. 26 patients were tested in our laboratory in the Center of Human Genetics in Regensburg, the remaining 21 patients in different laboratories in Germany, Austria, Italy and Latvia. We included patients with known causative mutations (likely pathogenic or pathogenic variants according to the ACMG classification [29]) in 5 different genes in our study cohort: *LIS1/PAFAH1B1* (*n* = 38), *DCX* (*n* = 5 males), *DYNC1H1* (*n* = 2), *TUBA1A* (*n* = 1) and *TUBG1* (*n* = 1) (see supplemental Table 2). All patients had characteristic radiologic findings of lissencephaly based on Magnet resonance imaging (MRI). The MR images were separately assessed for this study centrally and in a standardized manner by a neuroradiologist (G. S.) with longstanding experience in the diagnosis of cerebral malformations in pediatric patients.

### Statistical analysis

We compared the neurodevelopmental outcome in the two biggest genetic subgroups of patients with mutation in the *LIS1/PAFAH1B1* and *DCX* gene using the Mann-Whitney U test. Here the patients´ ability to always or sometimes reach certain milestones was contrasted with patients, who rarely or never show these abilities. We reported the asymptotic significance (two-tailed), a P value of < 0.05 was considered statistically significant. The effect size was calculated using Pearson´s correlation coefficient (*r*).

Analysing the HRQL and family functioning with the PedsQL™ Family Impact Module questionnaire, the 5-point response scale, the parents filled in, was transformed to a 0-100 scale in accordance with Varni et al. [27]. If separate questionnaires of both parents were available, the mean value was taken. In addition to the total score, which is made up of all 36 items, the parental HRQL summary score (made up of 20 items from the scales for physical, emotional, social and cognitive functioning) and the family functioning summary score (made up of 8 items from the scales for daily activities and family relationships) were calculated. Correlation between parental HRQL and seizure frequency was conducted using linear regression analysis. We determined an unadjusted P value of *p* < 0.05 to be significant (without multiplicity adjustment). A one-sample t-test was performed to assess the differences in quality of life between the families in our study and the data from a cohort of families without children with chronic diseases. A one-sided P value < 0.05 was considered significant. Effect sizes were expressed as Cohen´s *d*.

Efficacy of the various supportive therapies used in our study cohort was rated by the families, and the results were ranked in descending order based on the Kruskal-Wallis rank sum. Therapies rated in fewer than five patients were excluded from the analysis.

This study was exploratory in nature, aiming to identify potential trends rather than draw confirmatory conclusions, therefore, no adjustment for multiple testing was applied, in line with the recommendations of Bender and Lange [30].

Statistical analysis was performed with SPSS Software, version 26.0.

## Results

### Pregnancy and birth

#### *LIS1/PAFAH1B1* subgroup

Prenatally, 38% of the *LIS1/PAFAH1B1* patients (14/37) were found to have any abnormality, with microcephaly (7/37; 19%) being the most common finding observed on prenatal ultrasound. Birth mode was spontaneous vaginal delivery in 28/37 patients (76%) and 35/38 (92%) patients were born at term. All 10-minute APGAR scores were within normal range (31/31). Abnormal birth measures were observed in nearly half of the patients (15/33; 46%), with microcephaly in 12/31 (39%) and low birth weight for gestational age (SGA) in 7/36 (19%). Neonatal abnormalities including sucking weakness and muscular hypotonia were found in 18/37 (49%) patients (see Table 1). 8/33 neonates were treated in the neonatal intensive care unit (NICU) with a median duration of 7.5 days. A total of 71% of *LIS1* patients had at least one sibling. One sibling was reported to have had absence epilepsy in childhood. None of the other siblings in the *LIS1* cohort were diagnosed with any other severe chronic disease.

#### *DCX* subgroup

Prenatal abnormalities were observed in 2/5 of *DCX* patients (40%), with microcephaly in one patient (1/5; 20%). All patients were born at term with normal APGAR scores. One patient (1/3; 33%) exhibited microcephaly. Neonatal abnormalities were described in 2/5 (40%) individuals (see Table 1). Siblings were noted in 4/5 (80%) of patients. Epilepsy or neurodevelopmental compromise were reported in two mothers and three siblings (one sister diagnosed with subcortical band heterotopia) of the *DCX* patients. No further details on their phenotype or genetic result are available.

#### Others

The one *TUBA1A* patient was born at 39 weeks gestational age (GA) with a normal APGAR score and normal birth measures. Due to an abnormal prenatal ultrasound, a prenatal MRI had been performed confirmed the suspected diagnosis of lissencephaly. After birth, she was observed in the NICU for 6 days because of suspected neonatal seizures. The one *TUBG1* patient was born at 42 weeks GA, with an APGAR score and birth measures within normal range. Neonatal feeding and sucking difficulties were reported. The two *DYNC1H1* patients were delivered via caesarean section at term. 10-minute APGAR scores were normal. No prenatal abnormalities were noted in the *TUBG1* and the two *DYNC1H1* patients. All but one *DYNC1H1* patient were single children. No chronic condition was noted in that sibling.

**Table 1:** Clinical characteristics of *LIS1* and *DCX* patient cohorts

patient cohort	LIS1	DCX
number of patients	*n* = 38	*n* = 5
median age [years]	6.0	10.0
prenatal abnormalities	37.8% (14/37)	40.0% (2/5)
microcephaly	18.9% (7/37)	20.0% (1/5)
SGA	13.5% (5/37)	0.0% (0/5)
reduced fetal movements	10.8% (4/37)	0.0% (0/5)
enlarged ventricles	5.4% (2/37)	0.0% (0/5)
polyhydramnios	5.4% (2/37)	0.0% (0/5)
oligohydramnios	0.0% (0/37)	20.0% (1/5)
birth		
premature birth	7.9% (3/38)	0.0% (0/5)
birth mode: Sectio	24.3% (9/37)	25.0% (1/4)
normal APGAR score^1^	100% (31/31)	100% (3/3)
abnormal birth measures	45.5% (15/33)	50.0% (2/4)
microcephaly	38.7% (12/31)	33.3% (1/3)
SGA^2^	19.4% (7/36)	0% (0/3)
abnormalities during neonatal period	48.6% (18/37)	40.0% (2/5)
sucking weakness	40.5% (15/37)	40.0% (2/5)
muscular hypotonia	13.5% (5/37)	20.0% (1/5)
breathing problems	5.4% (2/37)	0.0% (0/5)
seizures	0.0% (0/37)	0.0% (0/5)
first clinical symptom		
seizure	46.2% (12/26)	(0/1)
neurodevelopmental delay	15.4% (4/26)	(1/1)
muscular hypotonia	15.4% (4/26)	(0/1)
Other	34.6% (9/26)	(0/1)
Median age when diagnosis suspected [months]	5.0^3^	9.0
neurologic assessment		
median age at assessment [years]	6.4	12.0
muscular hypotonia	92.9% (26/28)	0.0% (0/3)
spasticity	28.6% (8/28)	33.3% (1/3)
scoliosis	25.9% (7/27)	33.3% (1/3)
contractures	32.1% (9/28)	33.3% (1/3)
visual impairment	69.4% (25/36)	50.0% (2/4)
hearing loss	23.5% (8/34)	0.0% (0/4)
medical complications		
epilepsy	100% (38/38)	80.0% (4/5)
median onset of epilepsy [months]	6.0	5.0
complications^4^ related to food intake	62.2% (23/37)	50.0% (2/4)
PEG/ feeding tube	39.5% (15/38)	20.0% (1/5)
recurrent pulmonary infections	36.8% (14/38)	25.0% (1/4)

^1^APGAR score at 10 min ≥ 9

^2^birth weight < 3. percentile

^3^one patient with prenatal suspected diagnosis

^4^dysphagia/choking/vomiting

### Diagnosis & clinical evaluation

#### *LIS1/PAFAH1B1* subgroup

In the subgroup of 38 patients with *LIS1/PAFAH1B1* mutation, the median age at suspected diagnosis was 5 months. In one patient, an early diagnosis at 22 weeks GA was suspected in a routine prenatal ultrasound. Routine prenatal screening examinations were conducted in over half of the cohort (21/38; 55%), with suspect results observed in only 4/21 (19%) patients. First clinical signs or symptoms included most frequently epileptic seizures, neurodevelopmental delay, and feeding problems. Neuropediatric assessments at a median age of 6.4 years (range 0.5 to 24 years) revealed the presence of muscular hypotonia and visual impairment (see Table 1) as the most prominent symptoms.

#### *DCX* subgroup

Median age at suspected diagnosis for 4 of the 5 *DCX* patients was 9 months, one patient was diagnosed as late as 14 years of age. Prenatal screening examinations were performed in 3/5 (60%). Neurological assessments at a median age of 12 years showed spasticity, contractures and scoliosis in one patient each (1/3; 33%). Visual impairment was documented in half of the *DCX* patients (2/4) (see Table 1).

#### Others

First abnormalities in the *TUBA1A* patient were already recognized in a prenatal ultrasound screening, whereas the causative mutation in the *TUBA1A* gene was identified at the age of 3.5 months. Clinical assessment at the age of 3 months revealed generalized muscular hypotonia. In the individual with a *TUBG1* mutation, a clinical diagnosis was made at the age of five months after the onset of epileptic seizures and concomitant psychomotor retardation. Cerebral MRI revealed a thin cortex with enlarged lateral ventricles and partial pachygyria with a posterior-anterior gradient. At the age of 3.8 years, a pediatric neurological evaluation revealed muscular hypotonia. In the two patients with a mutation in *DYNC1H1*, the diagnosis of lissencephaly was suspected at 7 and 12 months, respectively. In a pediatric neurological clinical evaluation at the age of 13 months and 7 years, respectively, both were diagnosed with generalized muscular hypotonia as the only abnormality.

### Neurodevelopmental outcome

#### *LIS1/PAFAH1B1* subgroup

At the time of the study, the median age of the children was 6 years (range from 0.6 to 25 years). Approximately half of the children with *LIS1/PAFAH1B1* pathology achieved early developmental milestones (head control 24/36 (66.8%), babbling 20/34 (58.8%), rolling over 20/36 (55.6%), directed arm movements 14/36 (38.9%)). Achievement of more advanced developmental milestones, including pinch gripping, crawling, free sitting, standing without support and walking with support, was observed in less than 20% of children. Two patients in the cohort were described as having achieved free walking. Nearly all communicative milestones were absent. There was a follow-up subgroup with 14 participants, including nine patients with mutation in the *LIS1/PAFAH1B1* gene. Of those, six *LIS1/PAFAH1B1* patients were reported to have a partial regression of their abilities. 3/9 patients (33.4%) lost the ability to control their head, 2/9 (22.2%) the ability to grip an object, 2/9 (22.2%), the ability to roll over, 1/9 (11.1%) the function of visual fixation.

#### *DCX* subgroup

At the time of psychomotor evaluation, patients of the *DCX* subgroup had a median age of 10 years (range: 6.5 to 21.1 years). Four of these five patients (80%) achieved early infantile milestones (head control, rolling over, babbling), and three of five (60%) were able to directed arm movement, unsupported sitting and crawling. Two patients (2/5; 40%) were able to either speak single words and two-word sentences and demonstrated the ability to pinch grip, or to stand and walk without support, respectively.

#### Comparison of *LIS1/PAFAH1B1* and *DCX* cohort

**Fig. 1:**
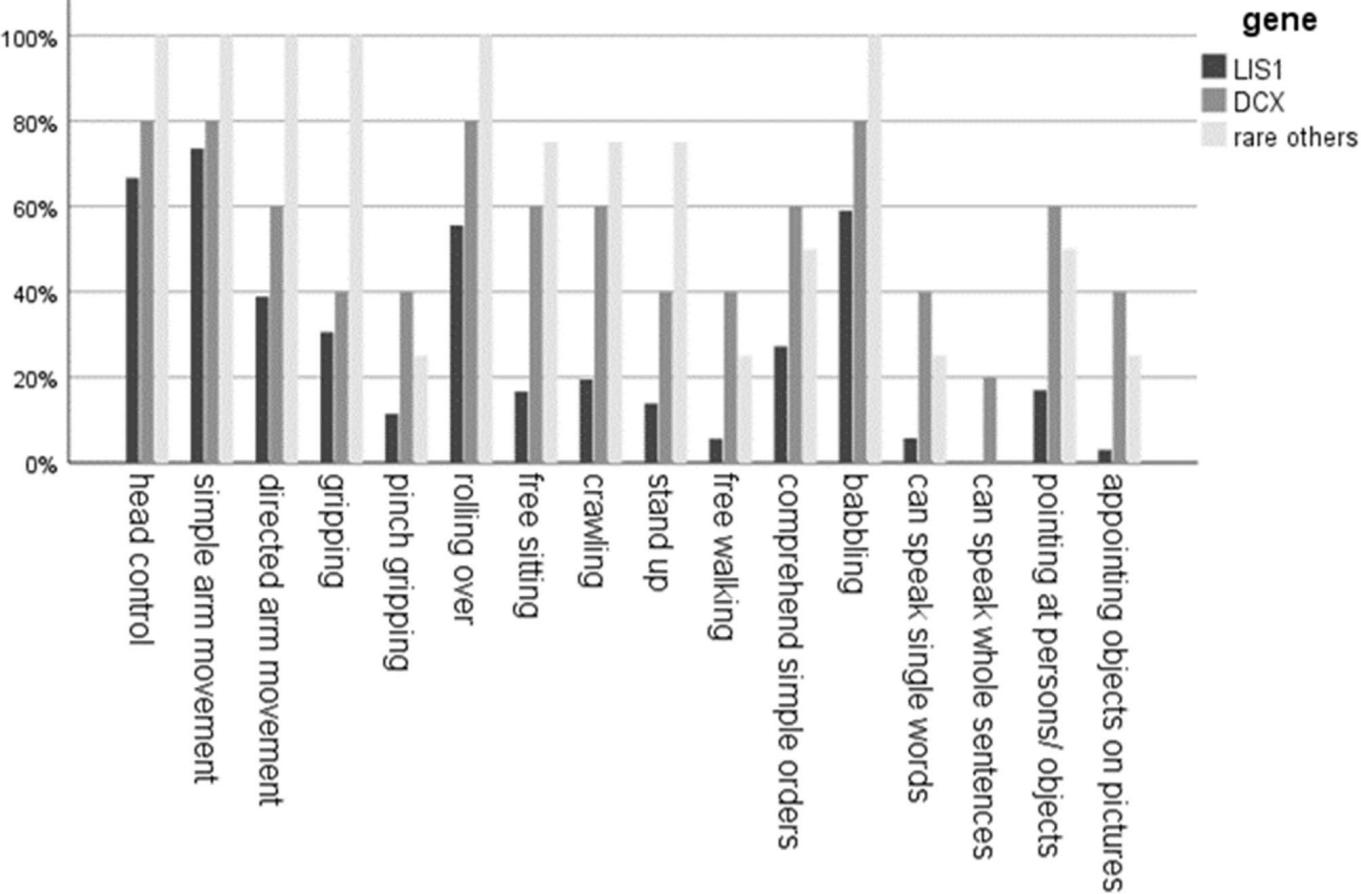
Neurodevelopmental outcome in three genetic subgroups: the *LIS1/PAFAH1B1* subgroup (*n* = 36), the *DCX* subgroup (*n* = 5), and rare others (*DYNC1H1*, *n* = 2; *TUBA1A*, *n* = 1; *TUBG1*, *n* = 1). Neurodevelopmental outcome was compared between the two largest genetic cohorts (*LIS1/PAFAH1B1* and *DCX)* using the Mann-Whitney U test. Asterisks (*) indicate statistical significance (*p* < 0.05)

As illustrated in Fig. 1, there are significant differences in the neurodevelopmental outcome between the *LIS1/PAFAH1B1* and the *DCX* cohorts. A significant higher proportion of *DCX* patients achieved more advanced milestones of development compared to *LIS1/PAFAH1B1* patients, including sitting without support (*z*=-2.17, *p* = 0.030, *r* = 0.34), free walking (*z*=-2.23, *p* = 0.026, *r* = 0.37), pointing at persons or objects (*z*=-1.97, *p* = 0.049, *r* = 0.32), speaking of single words (*z*=-2.184, *p* = 0.029, *r* = 0.36) and whole sentences (*z*=-2.53, *p* = 0.011, *r* = 0.42) and appointing objects on pictures (*z*=-2.77, *p* = 0.006, *r* = 0.46).

#### Others

At the time of data collection, the *TUBA1A* patient was 20 months old. She was able to control her head, turn around and perform directed arm movements. She could babble, but was unable to crawl, sit or stand without support or speak words. The other patients with *TUBG1* (at age 3.8 years) and *DYNC1H1* mutation (at age 5 and 8 years, respectively) had achieved the motor milestones of the first 9 months of life, such as head control, rolling over, sitting freely, pulling up to stand and crawling. In this “others” subgroup, free walking was observed in one *DYNC1H1* patient (starting at 32 months of age) and the *TUBG1* individual was able to walk with support. The milestones of cognitive development were more diverse among the different genetic subtypes; babbling was found in all four patients, further developmental steps such as speaking of single words have only been observed in the child with *TUBG1* mutation.

### Medical complications and survival

#### *LIS1/PAFAH1B1* subgroup

In all 38 children, epilepsy was diagnosed with a median age of onset of 6 months (range 2 months to 3.5 years). Feeding problems such as dysphagia, regurgitation, and vomiting were reported in 23/37 (62%) *LIS1/PAFAH1B1* patients. A gastrostomy/ feeding tube was present in 15/38 (40%) patients. 14/38 (37%) of patients had recurrent respiratory infections (minimum 1 pneumonia/ year). At the time of the study 5/38 *LIS1/PAFAH1B1* patients had died from unknown causes with a median age at death of 9.1 years. The oldest living patient was 25 years old.

#### *DCX* subgroup

Epilepsy was diagnosed in 4/5 (80%) males with a median age at onset of 5 months. Feeding problems were reported in half of the patients (2/4; 50%). One patient (1/5; 20%) had a feeding tube/ gastrostomy. Recurrent pneumonia was present in 1/4 (25%). At the time of data collection, one patient (1/5; 20%) had died at the age of 6.8 years, the cause is unknown.

#### Others

At the time of data collection, the girl with the *TUBA1A* mutation was 20 months old and has never had an epileptic seizure. She was fed orally and had frequent signs of dysphagia and regurgitation. Onset of epilepsy was documented at 5 months of age in the *TUBG1* patient and at 11 and 16 months, respectively, in the *DYNC1H1* patients. The *TUBG1* patient and one of the *DYNC1H1* patients were able to feed and eat independently without the need for a tube feeding. In the two *DYNC1H1* patients no feeding problems were reported, whereas the *TUBG1* patient had daily dysphagia and regurgitation. Pulmonary infections were absent in all patients in this “others” subgroup.

### Health-related quality of life

**Fig. 2:**
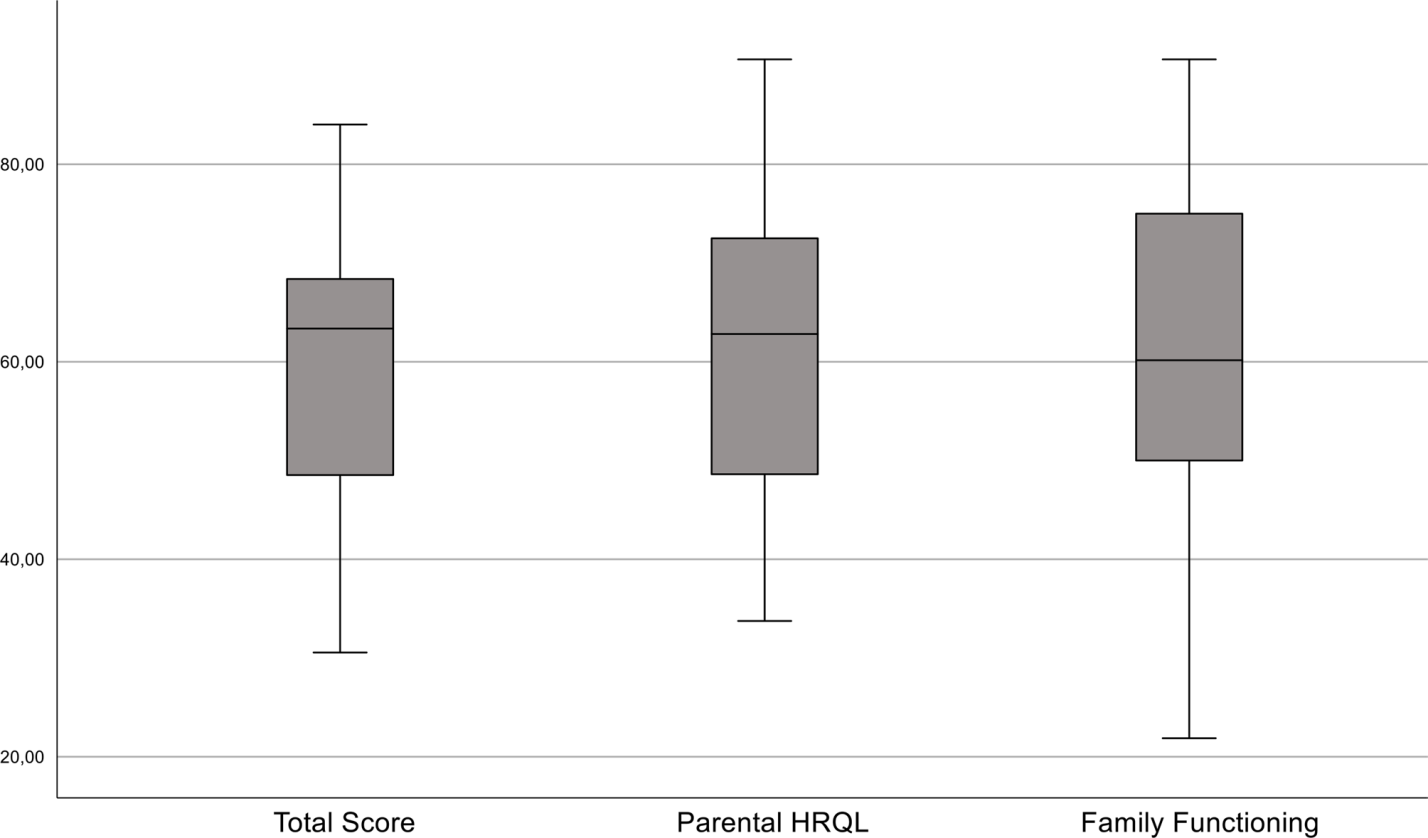
Health-related quality of life assessed using the PedsQL™ Family Impact Module in our lissencephaly patient cohort. Valid data were available for 18 patients

The mean HRQL total score was 58.54 (*n* = 18, SD 16.22), with the lowest (worst) scores recorded for the sections “worries” (46.94) and “daily activities” (43.39). The mean parental HRQL summary score was 61.23 (SD 16.79) and the mean family functioning summary score was 58.86 (SD 19.03) (Fig. 2). The parental HRQL score and the total score of the PedsQL™ Family Impact Module tended to be lower in families of children with frequent seizures, but did not reach statistical significance. Using a one-sample t-test (significance level *p* < 0.05) the HRQL Family Impact Module in our cohort was compared to the reference cohort by Medrano et al. [31], which excluded chronically ill children. Significant differences were observed for the total score (*p* < 0.001, *d=*-0.904), the parental HRQL summary score (*p* = 0.011, *d=*-0.594), and the family functioning summary score (*p* = 0.034, *d*=-0.460).

### Evaluation of supportive therapies

**Fig. 3:**
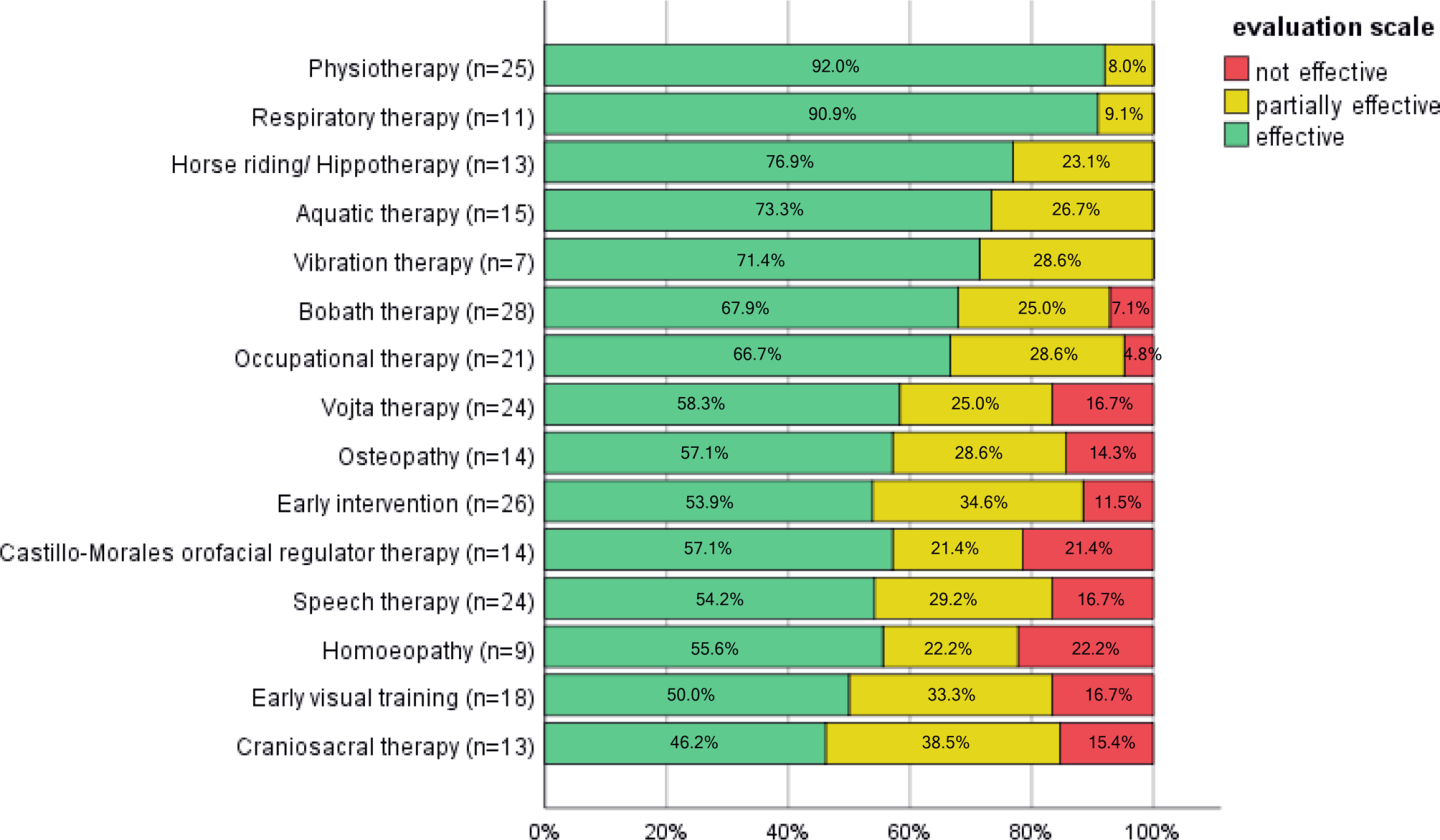
Efficacy of supportive therapies in patients with lissencephaly (*n* = 36), ranked in descending order based on the Kruskal-Wallis rank sum. Therapies were rated by the families and categorized as effective (green), partially effective (yellow), or not effective (red)

For 36/47 patients (28 *LIS1/PAFAH1B1* patients, 5 *DCX* patients, 1 *DYNC1H1*, 1 *TUBA1A*, 1 *TUBG1*), data on the applied supportive therapies used were available for evaluation. 1–17 different supportive therapies were used per patient, with a median of 7.5 different therapies per patient. The evaluated efficacy of the different supportive therapies varied considerably (see Fig. 3). Physiotherapy (*n* = 25) and respiratory therapy (*n* = 11) were ranked the most effective (100% effective), with good or excellent efficacy in more than 90% (physiotherapy 23/25; respiratory therapy 10/11). Horse riding/ hippotherapy (*n* = 13) and aquatic therapy (*n* = 15) were classified as excellent or of good efficacy in 10/13 (76.9%) and 11/15 (73.3%), respectively. Vibrational therapy (*n* = 7), Bobath therapy (*n* = 28), and occupational therapy (*n* = 21) were rated as having excellent or good efficacy in almost 70% of cases. In contrast, early visual training (*n* = 18) and craniosacral therapy (*n* = 13) were rated as less effective, showing no or partial effect in half of the patients (50.0% and 53.9%, respectively).

## Discussion

Lissencephaly is well recognized as a severe brain malformation resulting in early epilepsy, muscular hypotonia, severe neurodevelopmental delay and increased childhood mortality [21, 23, 32]. In this present study we describe in more detail the severe neurodevelopmental phenotypic spectrum of 47 patients with genetically confirmed lissencephaly including various medical complications and long-term course and its profound impact on daily quality of life of their families. Our data provide significant novel data for medical care givers and guide information of the families newly confronted with this diagnosis in their children.

### Pregnancy, birth and infancy

The prenatal diagnosis of lissencephaly is challenging. In this cohort only a minority of patients were identified during routine prenatal examinations to exhibit sonographic abnormalities. The most prevalent prenatal abnormality identified was fetal microcephaly, which occurred in approximately 20% of the *LIS1/PAFAH1B1* subgroup and the *DCX* subgroup, respectively. Two patients of the *LIS1/PAFAH1B1* subgroup were diagnosed with dilated ventricles in prenatal ultrasound. Both findings are considered “red flags” and could lead to further diagnostic workup including prenatal MRI and genetic testing [7, 8].

It is noteworthy that, in the overall cohort, irrespective of genotype, the prematurity rate and the ceasarian section rate was not elevated, and APGAR scores were normal in all neonates. As previously reported, neonatal birth measurements could serve as a potential diagnostic indicator in a subset of patients [32, 33]. In our study, microcephaly at birth was observed in 39% of *LIS1/PAFAH1B1* patients and 50% of *DCX* patients. Low birth weight for gestational age was noted in 19% of the *LIS1/PAFAH1B1* subgroup. Furthermore, approximately half of the neonates exhibited clinical signs of muscular hypotonia and weak sucking, with some requiring treatment in a neonatal intensive care unit (NICU).

During infancy, the clinical key features – epileptic seizures, developmental delay, and muscular hypotonia – typically occur in most patients, leading to MR brain imaging and genetic diagnostics [4, 21, 33]. In our cohort, diagnosis of lissencephaly was suspected at a median age of 5 months in the *LIS1/PAFAH1B1* subgroup and 9 months in the *DCX* subgroup. The diagnostic age range was wide, spanning from prenatal to 14 years of age in individual cases. Recommendations for the clinical approach to genetic testing have been published before [3, 18].

### Neurodevelopmental outcome

A profound developmental delay is a hallmark of lissencephaly patients [4, 20, 21, 33, 34]. Accordingly, all patients of our study displayed more or less severe developmental disability. At the most severe end of the spectrum, one third of the children with mutation in the *LIS1/PAFAH1B1* gene did not even achieve head control. In this *LIS1/PAFAH1B1* subgroup, only milestones of the first months of life, such as visual fixation (85%), social smiling (69%), head control (67%) and babbling (59%) were achieved by the majority of children. In contrast, hardly any of these patients mastered more advanced milestones of the second half of the first year of life, with less than 20% of children achieving sitting (6/36), crawling (7/36) and walking with support (5/32). Two patients in the present *LIS1/PAFAH1B1* cohort were reported to walk independently. These findings were consistent with previous evaluation of our Regensburg *LIS1/PAFAH1B1* cohort, published in 2016 [20], and with previously published reports of other cohorts [23, 33, 34]. As in previously published reports we also observed individual patients with apparently milder phenotype in the presence of a *LIS1/PAFAH1B1* mutation [1, 5, 18].

In our study, patients with *DCX*-associated lissencephaly were more likely to achieve more complex milestones of development in comparison to patients with *LIS1/PAFAH1B1*-associated lissencephaly (Fig. 1). This was significant *(p* < 0.05*)* for achieving independent walking and speaking single words or whole sentences. This finding is in line with other previously published cohorts of patients with *DCX*-related lissencephaly [24, 25]. For example, 40% (2/5) of the *DCX* cohort in this study were able to walk unaided, in agreement with Leger et al., who described the ability to walk independently in a percentage of 46% in a *DCX* cohort of 33 patients [24].

In this study, a follow-up questionnaire was available from a subgroup of patients (*n* = 14), which made it possible to formulate new statements about the long-term course of the disease. A notable finding in this regard was the observation of developmental regression in some patients, with the loss of abilities (for example the loss of visual fixation, head control or gripping in advanced age) that had been previously acquired. This finding was reported in parallel with a deterioration in the nutritional status associated with worsening epilepsy over time [21] and suggests that psychomotor regression might be a consequence of drug-resistant epilepsy and long-term ASM (antiseizure medication) therapy, especially considering that the vast majority of patients in the present study cohort experienced epileptic seizures.

In our present study, the data on the development of the subgroup “rare others” with a causative mutation in *DYNC1H1*,* TUBA1A* or *TUBG1* were heterogeneous and must be interpreted with caution due to the very limited number of patients. All 4 patients were moderately to severely impaired in various developmental domains. Previously published data from patients with a *DYNC1H1*-associated neuronal migration disorder likewise described intellectual disability, psychomotor retardation and neurological findings, but with varying degrees of severity [22, 35, 36].

Most data on *TUBA1A* lissencephaly focus on MRI findings correlated with the mutation, fewer data are available on the clinical course. Previously reported lissencephaly patients with *TUBA1A* mutations also presented with severe developmental delay and impairment [37–40]. However, also milder phenotypes of patients with *TUBA1A-*associated cerebral malformations other than classic lissencephaly were described [41]. At study enrollment at 5 months of age, the female with *TUBA1A* mutation of our study presented with generalized muscular hypotonia but no developmental delay and no epileptic seizures at this early age. It will be interesting to further evaluate the future course of disease.

Clinical data on very rare *TUBG1*-associated lissencephaly include posterior predominant pachygyria, microcephaly and an often severe clinical phenotype including spastic quadriplegia and early onset epilepsy [16, 42–44]. Of note, Brock et al. collected information on eight patients with mutation in the *TUBG1* gene, five of whom had a mutation at the same locus as the patient of this study [43]. Not surprisingly, the patient in the present study showed similar clinical and MRI features and epilepsy of early onset, but managed to achieve some motor development with walking with support.

In summary, although the small number of patients in the “rare others” subgroup precluded statistical evaluation, the descriptions of the developmental and epileptic phenotypes added valuable data to the medical literature that may be helpful in counselling other affected families.

### Medical complications and survival

Epilepsy, frequently drug-resistant, is another hallmark of lissencephaly patients [45]. Epilepsy was diagnosed in 100% of *LIS1/PAFAH1B1* patients and 80% of *DCX* patients in this study. The age at seizure onset was similarly early in the two main subgroups, with a median age of 6 months in the *LIS1/PAFAH1B1* and 5 months in the *DCX* subgroup, which is in line with previous reports [21, 33, 34, 46]. Furthermore, a comparable daily seizure burden was initially observed in the *LIS1/PAFAH1B1* patients (median 1.0 seizures per day) and the *DCX* patients (median 2.0 seizures per day). A detailed and comprehensive analysis of epilepsy, seizure types and response to antiseizure medication for our cohort was published elsewhere [20]. In addition to epilepsy, we here describe in detail a number of other common medical problems in these lissencephaly patients, including dysphagia, regurgitation, tube feeding requirements, and recurrent respiratory infections and pneumonia [21]. Complications related to food intake were more prevalent in our *LIS1/PAFAH1B1* subgroup when compared to the *DCX* subgroup. This was evidenced by the presence of a gastrostomy in 40% of *LIS1/PAFAH1B1* patients and 20% of *DCX* patients, and frequent dysphagia in 62% of *LIS1/PAFAH1B1* patients and 50% of *DCX* patients. These findings suggest that the more severe neurodevelopmental condition observed in *LIS1/PAFAH1B1* patients may be a contributing factor.

In contrast to the very poor survival in classic lissencephaly patients described in previous reports from 2010 to 1991 [2, 21], survival was notably increased in the present study population. In the current era of improved medical care including aggressive infection management, routine gastrostomy and respiratory therapy, a significant proportion of patients with classic lissencephaly can reach the age of 20 years or even older. Likewise, an increased life expectancy had been reported in patients with *LIS1/PAFAH1B1*-related lissencephaly [45], in this study population 7/38 patients in the *LIS1* cohort (18%) were older than 15 years at the time of examination.

### Supportive therapies

For the first time, data on the use of supportive therapies in patients with lissencephaly were collected and the effectiveness of the different therapies was assessed on a subjective parental basis. Many families rated the therapies used as good or excellent, with respiratory therapy and physiotherapy ranked best. Similarly, a case of a 2-year-old child with lissencephaly reported improvements in various aspects such as muscle tone, assessed on an individual and subjective basis, after a 12-week program of daily application of different physiotherapy rehabilitation techniques [47]. In children with cerebral palsy, hippotherapy was found to be beneficial in physical, cognitive and social aspects [48]. However, meta-analyses of intervention programs in children with different neurodevelopmental disorders often complain about the lack of high-quality studies and show limited convincing conclusions about the efficacy of these therapies [49]. Yet, it could be hypothesized that the different therapists themselves play a central role for the patients and especially their families by providing understanding of current problems and formulating future therapy goals to work on together. This may serve as motivation and be perceived as support for the caregivers on a subjective basis.

An important finding of our study was that variable supportive therapies are widely used in patients with lissencephaly, with a median of 7.5 different therapies used overall per patient. This reflects tremendous efforts of caregivers to support their children in all aspects and to do everything possible to improve not only the medical situation, but also overall quality of life. For the patients in our study, the wide range of supportive therapies was often reported as easily accessible thanks to specialized outpatient clinics (Sozialpaediatrische Zentren SPZ), a German facility for the care of chronically neurologically ill children.

### Quality of life

Severe and often rare chronic neurological diseases in childhood place a heavy burden on caregivers and the broader family system [50, 51]. To date, there is no data on the quality of life of patients with lissencephaly, nor on the impact on the family and the quality of life of parents caring for affected patients. The present study found significantly (*p* < 0.05) reduced scores for health-related quality of life and family functioning compared to reference data from families with healthy children [31]. Similarly, significantly reduced values for PedsQL™ total score (mean 57.3; SD 17.5), parental HRQL (60.8; 18.3) and family functioning (49.8; 22.2) were also reported in families with metachromatic leukodystrophy and pontocerebellar hypoplasia type 2, which may serve as examples of other rare and very severe pediatric neurological disorders [50]. However, in other reports of families with severely disabled children after a near-drowning, the health-related quality of life of the parents was not impaired compared to the normal population, which is inconsistent with our data [52]. Furthermore, in families with children diagnosed with various types of epilepsy, a significant negative impact on the parents’ quality of life and family functioning was described before [53]. When analyzing the sub-domains, in line with our study, the best scores were obtained in the domains of “cognitive functioning” and “family relationships”, and the worst scores were obtained in the domains of “worry” and “daily activities”, reflecting parents’ well-founded concerns about their children’s health and future prospects and the resulting limitations in daily activities. The present study showed a non-significant trend towards an inverse relationship between parents’ quality of life and seizure frequency, with a better quality of life being associated with a lower seizure burden. This parallels the quality of life data from epilepsy patients, specifically with Dravet syndrome and Lennox-Gastaut syndrome, where lower seizure frequency and higher number of seizure-free days correlated with better quality of life [54–56]. These observations underscore that treating and improving epilepsy may have a significant impact on the quality of life of the whole family and should be at the center of the therapeutic efforts of the treating physicians.

### Limitations

In addition to the retrospective design of this study, the most obvious limitation is that still only a small number of patients with causative mutations in genes other than *LIS1/PAFAH1B1* could be included. Due to the very limited sample size, the four patients of the “rare others” subgroup must be regarded as individual case reports and were consequently excluded from the comparative analysis of the genetic subgroups. However, these diseases are extremely rare, and despite the involvement of several study centers, only this limited number of individuals could be diagnosed and included. As recruitment was not random and some of the patients were recruited through collaboration with a lissencephaly self-support group (Liss e.V.), participating families may have been specifically motivated to care for their children. Due to their young age at the time of the study, some of the patients had to be excluded from some assessments, such as advanced motor milestones. A strength of this study is that the participating patients were characterized in great detail and the study covered a wide age range. Some follow-up examinations were also possible. In addition, for the first time, there are some new data on parental quality of life, the burden of the disease on the family, and the use and parental evaluation of supportive therapies which may suggest potential effects on the disease course.

## Conclusion

- Individuals with lissencephaly present with a variable but mainly severe phenotype that is highly dependent on the affected gene that causes lissencephaly.
- Prenatal diagnosis of lissencephaly remains challenging and the majority of patients with genetically confirmed forms of lissencephaly will only be detected postnatally, usually after an inconspicuous neonatal period, as abnormal cortical migration often is not detected at the time of routine prenatal ultrasound screening.
- Clinically, the combined observation of muscular hypotonia, developmental delay, microcephaly and epilepsy remains the clue for diagnostic confirmation by imaging and genetic analysis for the majority of patients.
- Patients with *LIS1/PAFAH1B1* -related lissencephaly have a significantly worse psychomotor prognosis than *DCX-*related lissencephaly.
- Apart from pediatric neurological complications, respiratory and gastrointestinal problems are very common.
- Supportive therapies are used intensively by affected families and are very often rated as excellent or good on a subjective basis.
- Lissencephaly is a very severe chronic progressive pediatric neurological disorder placing a high burden on caregivers and the entire family, which may relate to seizure activity in patients. Our data confirm the central role of effective treatment of the epilepsy and multi-professional support on quality of life of these patients and their families.

## Electronic Supplementary Material

Below is the link to the electronic supplementary material.

Supplementary Material 1

Supplementary Material 2

Supplementary Material 3

## Data Availability

The datasets used and analysed during the current study are available from the corresponding author on reasonable request.
