# Supplementary material for: Gene-specific long-term course, neurodevelopmental outcome and quality of life in patients with LIS1/PAFAH1B1-, DCX-, DYNC1H1-, TUBA1A- and TUBG1-related lissencephaly
Source: Orphanet J Rare Dis. 2026 May 23;21:206. doi: 10.1186/s13023-026-04398-z (PMC13202861; doi:10.1186/s13023-026-04398-z)
Supplement: Supplementary file 1 — Supplementary Material 1 [file 13023_2026_4398_MOESM1_ESM.pdf]

Questionnaire for doctors
Study of the long term progression of the lissencephaly afflicted

Study-ID: _________ **Birthdate:** ___/___/_______

1. DIAGNOSTIC

When was the lissencephaly (tentatively) diagnosed?

○ Prenatal O Perinatal O Postnatal (at the age of_______________)

First clinical Symptom:

____

Which diagnostic tests were performed?

O CT O MRT/MRI O cranial ultrasound
O prenatal MRT O prenatal ultrasound O gene test
O TORCH-serology: (O abnormal, because ………………………………………………)

EEG Results: O not performed
O normal (Age: ……..… years) O abnormal (from Age: ……….. years):
O Hypsarrhythmia O focal Epileptiform activity
O generalized Epileptiform activity O other:……………

2. EPILEPSY AND ANTICONVULSIVE THERAPY

Seizures: O no (never) O yes, since ___/___ (month/year)

O BNS-Seizure (at age ….…… until …..) O Myoclonus (at age ….…… until ……...)
O absence (….…….. until ….……..) O gen. tonic-clonic seizure (….…….. until …..…)
O tonic seizure (….…….. until ……....) O complex-focal seizure (…..…...until …..….)
O clonic seizure (........until ………...) O febrile seizure (………..until ……….)

Age during the first seizure: ……………… (Age in month/years)
Current number of seizures/time: ………………………….
Current nature of the seizure:………………………………………………………………………………
Current medication:………………………………………………………………………………………....

Response to anticonvulsive therapy:

Drug	Seizure decrease >50%	Seizure decrease >25%	Seizure decrease <25%	Clinical deterioration	Unusual side effects
Valproat	O	O	O	O	O
Topiramat	O	O	O	O	O
Phenobarbital	O	O	O	O	O
Phenytoin	O	O	O	O	O
Vigabatrin	O	O	O	O	O
Carbamazepin	O	O	O	O	O
Oxcarbazepin	O	O	O	O	O
ketogenic diet	O	O	O	O	O
Sultiam	O	O	O	O	O
Levetiracetam	O	O	O	O	O
Lamotrigin	O	O	O	O	O
Clonazepam	O	O	O	O	O
other:	O	O	O	O	O

Unusual side effects:

Drug Side effect

………………………………… ………………………………………………………………………………………

………………………………… ………………………………………………………………………………………

………………………………… ………………………………………………………………………………………

………………………………… ………………………………………………………………………………………

………………………………… ………………………………………………………………………………………

3. CURRENT CLINICAL STATUS

at the age of _____month____years

Circumference of the head _________cm weight____ kilograms Height_____cm

Best Motoric Performance:

O control of the head O unsupported sitting O crawling/rolling

O supported standing O supported walking O unsupported walking

Dysmorphia/abnormalities:

Skin (naevi, elasticity,…) O no/ O yes:____

Skull/face O no/ O yes:____

Thorax/ extremities O no/ O yes:____

Gastrointestinal/genitourinary O no/ O yes:____

Heart/Lung O no/ O yes:____

Vascular anomalies O no/ O yes:____

Clotting disorder O no/ O yes:____

Hearing impairment O no/ O yes:____

Sight impairment O no/ O yes:____

Other impariments: O no/ O yes:____

Study-ID: _________

Musclehypotonus: O lower extremity O upper extremity O trunk O generalized
O proximally accentuated O distally accentuated O proximal=distal

Contractures O none O Finger O wrist O elbow
O shoulder O ankle O knee O hip

Tremor O no O yes: _________

Spastic movement disorder O no O yes O hemiparesis right / left O tetraparesis
(upper extremity > = < lower extremity)

Eye movement: O directed O undirected O nystagmus O blind

Social interaction O normal O reaction to being spoken to O reaction to tactile stimulus O none

Endocrinology

Have any of the following ever been observed (even briefly?)

O diabetes insipidus O diabetes mellitus O growth hormone deficit
O hypo/ hypercortisolism O hypo/hyperthyroidism O electrolyte derailment

Does your patient suffer from respiratory ailments, dyspnea, or a predisposition for pneumonia?

____

4. COMPLICATIONS

Hospitalizations /operations? If available, please provide a copy of the discharge summary

Year Reason/operation

…….. ……………………………………………………………………………………………………..
…….. ……………………………………………………………………………………………………..
…….. ……………………………………………………………………………………………………..
…….. ……………………………………………………………………………………………………..
…….. ……………………………………………………………………………………………………..

O **Death of Patient**

- At the Age of____
- Cause of death____
- Was reanimation attempted O yes (how often) O no
- Was an autopsy performed? O yes O no

Attachments O medical reports O EEG results O MRT/MRI Pictures (CD copy or film)

____
Doctors Name

Stamp of the doctor or clinic:

____
Location and date

____
Doctor's Signature

Study-ID: _________
