# Supplementary material for: Gene-specific long-term course, neurodevelopmental outcome and quality of life in patients with LIS1/PAFAH1B1-, DCX-, DYNC1H1-, TUBA1A- and TUBG1-related lissencephaly
Source: Orphanet J Rare Dis. 2026 May 23;21:206. doi: 10.1186/s13023-026-04398-z (PMC13202861; doi:10.1186/s13023-026-04398-z)
Supplement: Supplementary file 2 — Supplementary Material 2 [file 13023_2026_4398_MOESM2_ESM.pdf]

Questionnaire for Families

Study of the long term progression of the lissencephaly afflicted

Study-ID:____________ **Birthdate:___/___/____**

1. Family, Pregnancy, and Birth

Mothers age at the birth of the child: ………………………………………….

Chronic illnesses of the mother: …………………………………………

Father's age at the birth of the child: ………………………………………….

Chronic illnesses of the father: ……………………………………………

Did you suffer a spontaneous abortion or a stillbirth?

- Yes : _______________in the ___ week of pregnancy.

Siblings:

			Healthy? If applicable, please specify their illness:
Age:___	male O	female O	yes O no,………………………………………………………....
Age:___	male O	female O	yes O no,………………………………………………………....
Age:___	male O	female O	yes O no,………………………………………………………....

Are there any other family members with a developmental disorder or who have suffered a spontaneous abortion?

………

………

If yes, please specify their relationship to the individual afflicted with lissencephaly

……

Peculiarities during the pregnancy, please include trivialities and feelings:

O None
O Infection
O Baby was too small
O decreased child movement
O head circumference was O too big O too small
O increased amniotic fluid (Polyhydramnios)
O other...

Illnesses of the mother during the pregnancy:

O Pregnancy diabetes
O increased liver values
O increased cholesterol
O Anemia
O Iron deficiency
O Seizures/Epilepsy
O Infection(s) (which?..)

Lab tests during the pregnancy (if applicable, please attach a copy):

O normal
O abnormal, because___________________________________

Environmental factors during the pregnancy

O Medication
O Alcohol
O Cigarettes
O Drugs
O if applicable, when/how long during the pregnancy

....

O vegetarian/vegan diet during the pregnancy? If yes, which:…………………………………………..

O other risk factors, i.e. environmental factors, plane travel: ....

Prenatal diagnostic (before birth)

Nuchal translucency	O normal	O abnormal	O was not performed
Triple test	O normal	O abnormal	O was not performed
Amniocentesis	O normal	O abnormal	O was not performed
Special Ultrasound	O normal	O abnormal	O was not performed
MRI	O normal	O abnormal	O was not performed

Birth

O on the due date　O _____ days before　O _________days after the due date

O spontaneous　O labor was medically induced　O Cesarean section

O emergency Cesarean section　O premature rupture of the membranes

Measurements at birth:

APGAR Scale: …../…../….. Weight (g): ……………　Length (cm): ………………

Head circumference (cm): ………………..

Hospital stay after birth ……….. Days:　O normal ward　O intensive care

Abnormalities after birth:

O Feeding problems　O Generalized muscle weakness　O External feeding tube

O Infection　O Breathing problems　O Artificial respiration

O Heart rhythm disorder　O Seizure

Other abnormalities:

……….

……

2. DIAGNOSIS

When was a lissencephaly tentatively diagnosed?

- Before the birth
- Within the child's first 6 weeks
- Later, at the age of ……………………………………………

Does your child suffer from other illnesses, or malformation of other organs?

O No　O Yes, ……………………………………………………………………………………..

Does your child suffer from a hearing or sight impairment?

O No　O Yes, ……………………………………………………………………………………..

Does your child suffer from breathing problems, or does he/she get lung infections easily?

O No　O Yes, ……………………………………………………………………………………..

How well did the doctor that told you of the diagnosis look after you?

O Very well　O Good　O Satisfactory　O Sufficient　O Unsatisfactory　O Inadequate

Studien-ID:

3. MEDICAL CARE

Food intake: O independent O fed O external feeding tube
Swallowing impairement: O daily O > 1x/week O > 1x/month O rarely/never
Vomiting while eating: O daily O > 1x/week O > 1x/month O rarely/never
Gagging while eating: O daily O > 1x/week O > 1x/month O rarely/never
Lung infection O > than 4x/year O 2-3x/year O 1x/ year O never

Do you have or did you ever have a home monitor for your child?
O Yes, from ………. (Age) until ……….. (Age) O No
Is/was this home monitor helpful?
O Yes O No

Age during the first seizure:……………. (age in months/years)
Situation during which the first seizure occured (for example, after a vaccination, during a fever, at night):
……….
………..

Number of seizures at the beginning: …………………………… (For example, 5 times daily, once a month, etc.)
Current number of seizures: ……………………………….

What medication did your child receive?
In your opinion, how well did your child respond to the medication?

Drug	Seizure decrease >50%	Seizure decrease >25%	Seizure decrease <25%	Clinical deterioration	Unusual side effects
Valproat	O	O	O	O	O
Topiramat	O	O	O	O	O
Phenobarbital	O	O	O	O	O
Phenytoin	O	O	O	O	O
Vigabatrin	O	O	O	O	O
Carbamazepin	O	O	O	O	O
Oxcarbazepin	O	O	O	O	O
Ketogenic diet	O	O	O	O	O
Sultiam	O	O	O	O	O
Levetiracetam	O	O	O	O	O
Lamotrigin	O	O	O	O	O
Clonazepam	O	O	O	O	O
Anderes:	O	O	O	O	O

Unusual side effects:

Drug Side effect
……………………………………… ……………………………………………………………………………………..
……………………………………… ……………………………………………………………………………………..
……………………………………… ……………………………………………………………………………………..
……………………………………… ……………………………………………………………………………………..

Studien-ID:

……………………………………… ………………………………………………………………………………………………..

Which combination of drugs worked the best, in your opinion?

……

4. DEVELOPMENT Current age:…………………………..

MOTOR FUNCTION	always	sometimes	rarely	never
Can control head movements	O	O	O	O
Can turn head to the side	O	O	O	O
Can nod	O	O	O	O
Can perform simple movements of the arms	O	O	O	O
Can perform precise arm movements	O	O	O	O
Can independently reach for an object	O	O	O	O
- With one hand	O	O	O	O
- With both hands	O	O	O	O
Can hold an object that has been given to him/her	O	O	O	O
- With one hand	O	O	O	O
- With both hands	O	O	O	O
Can pinch an object with two fingers	O	O	O	O
Can draw with a marker	O	O	O	O
- alone	O	O	O	O
- With help	O	O	O	O
Can point to an object	O	O	O	O
- With a hand	O	O	O	O
- With a finger	O	O	O	O
- With both hands	O	O	O	O
Can use a push-button	O	O	O	O
- alone	O	O	O	O
- with help	O	O	O	O
Can use multiple push-buttons	O	O	O	O
- alone	O	O	O	O
- With help	O	O	O	O
Can turn over by himself/herself	O	O	O	O
Can sit by himself/herself	O	O	O	O
Can crawl	O	O	O	O
Can pull himself/herself up	O	O	O	O
Can stand by himself/herself	O	O	O	O
Can walk with help (walker/parents)	O	O	O	O
Can walk by himself/herself	O	O	O	O

PERCEPTION AND COMMUNICATION	always	sometimes	rarely	never
Can focus on a person/object	O	O	O	O
Can follow a person/object	O	O	O	O
Can recognize a person/object	O	O	O	O
Can recognize a person in a photo	O	O	O	O
Can point to his/her own body parte (nose, ear, etc.)	O	O	O	O
Shows a reaction to his/her name	O	O	O	O
Can tell the difference between right and left	O	O	O	O
Can tell the difference between up and down	O	O	O	O
Can understand individual words	O	O	O	O
Can understand individual sentences	O	O	O	O
Can understand commands	O	O	O	O
Points to people/objects	O	O	O	O
Can mumble	O	O	O	O
sounds („mama, dada“)	O	O	O	O
Can speak individual words	O	O	O	O
Can say two word sentences („Mama gone“)	O	O	O	O
Can name a person	O	O	O	O
Can name an object in a picture	O	O	O	O
Can clearly say yes/no	O	O	O	O
Can speak in complete sentences	O	O	O	O
Reacts to music	O	O	O	O
Reacts to well-known voices	O	O	O	O
Reacts to animals	O	O	O	O
Contact smile (smiles when being spoken to)	O	O	O	O

What are your child’s strengths?

What hobbies or interests does your child have? What helps your child?

When were the first abnormalities in your child’s development noticed?

What do you find the most difficult?

How could one make this easier? In what aspects do you want and need more help?

Studien-ID:

5. EFFECTIVNESS OF SUPPORTING MEASURES

Does your child visit a nursery, kindergarten or school?

O No, our child is looked after at home From age:____________________

O at home nurse From age:____________________

O Nursery/Nanny From age:____________________

O Kindergarten From age:____________________

O other:_____________________ From age _____________________

What sort of establishment does your child attend?

O Counseling center O integrative establishment O Normal school/kindergarten

Please consider how helpful the following therapies were for your child (if attended):

TYPE OF THERAPY	From Age	Very effective	Effective	Somewhat effective	Not effective	harmful
Early counseling		O	O	O	O	O
Physiotherapy following Bobath		O	O	O	O	O
Physiotherapy following Voijta		O	O	O	O	O
Cranial-Sacral Therapy		O	O	O	O	O
Osteopathy		O	O	O	O	O
Training on a vibration plate (i.e. Galileo, Power Plate)		O	O	O	O	O
Swim therapy		O	O	O	O	O
Dolphin therapy		O	O	O	O	O
Riding or hippo therapy		O	O	O	O	O
Other animal therapy		O	O	O	O	O
Ergo therapy (work therapy)		O	O	O	O	O
Logopedics		O	O	O	O	O
Physical therapy		O	O	O	O	O
Food therapy after Castillo-Morales		O	O	O	O	O
Breathing therapy		O	O	O	O	O
Sight/blind counseling		O	O	O	O	O
Petoe therapy		O	O	O	O	O
Kinesiology		O	O	O	O	O
Homeopathy		O	O	O	O	O
Bach flower therapy		O	O	O	O	O
other:		O	O	O	O	O

Which remedy of the homeopathy or Bach flower therapy were effective/harmful?

THANK YOU VERY MUCH FOR YOUR PARTICIPATION!

Studien-ID:
