# Supplementary material for: Gene-specific long-term course, neurodevelopmental outcome and quality of life in patients with LIS1/PAFAH1B1-, DCX-, DYNC1H1-, TUBA1A- and TUBG1-related lissencephaly
Source: Orphanet J Rare Dis. 2026 May 23;21:206. doi: 10.1186/s13023-026-04398-z (PMC13202861; doi:10.1186/s13023-026-04398-z)
Supplement: Supplementary file 3 — Supplementary Material 3 [file 13023_2026_4398_MOESM3_ESM.docx]

**Supplemental Table 2: Genetic, radiological and clinical data of our lissencephaly patient cohort**

MRI Grading was performed according to the LIS-SBH-classification system by DiDonato et. al [17]

Gradient of gyral malformation: diffuse (GD), anterior more severe than posterior (GAP), posterior more severe than anterior (GPA), temporal more severe than posterior and P>A (GT)

Grade of gyral malformation: LIS partial pachygyria (LPP), LIS diffuse pachygyria (LDP), LIS agyria-pachygyria (LAP), LIS diffuse agyria (LDA)

Miller-Dieker-Syndrome (*PAFAH1B1*^+^*)*

n/a: data not available/ MRI was not available for Grading. m/f: male/female

| **ID (Sex)** | **Gene** | **Mutation (type)** | **Inheritance** | **MRI Grade/ Subtype, Gradient** | **Best motor skill** |
| --- | --- | --- | --- | --- | --- |
| P01 (m) | *PAFAH1B1* | Deletion Exon 3 to 3‘-UTR and locus *KIAA0664* | n/a | 3-2, GPA, LAP | Head control |
| P02 (m) | *PAFAH1B1* | Whole gene deletion until the *GARNL4* gene | De novo | n/a | Head control |
| P03 (m) | *PAFAH1B1* | Deletion exon 6-7 (6.2 kb) | De novo | 3-1, GPA, LAP | Holding onto an object |
| P04 (f) | *PAFAH1B1* | c.1066T>C; p.Cys356Arg | De novo | n/a | Operating a single key |
| P05 (m) | *PAFAH1B1* | IVS6-10T>C | De novo | 3-1, GPA, LAP | Rolling over |
| P06 (f) | *PAFAH1B1* | c.900+1G>A | n/a | n/a | Rolling over |
| P07 (m) | *PAFAH1B1* | c.58C>A; p.Arg20Ser | De novo | 3-1, GPA, LAP | Walking with support |
| P08 (m) | *PAFAH1B1^+^* | Microdeletion 17p13.3 | n/a | n/a | Rolling over |
| P09 (m) | *PAFAH1B1^+^* | Deletion 17p13.3 and subtelomer region 17pter | n/a | n/a | None |
| P10 (f) | *PAFAH1B1^+^* | Microdeletion 17p13.3 incl. *YWHAE* | n/a | n/a | Rolling over |
| P11 (m) | *PAFAH1B1* | Del ex8-9 | De novo | n/a | Head movement to side |
| P12 (m) | *PAFAH1B1* | Complex duplication in *PAFAH1B1-*critical region 17p13.3 incl. *YWHAE* | De novo | 3-1, GPA, LAP | Simple arm movement |
| P13 (m) | *PAFAH1B1* | c.282delG; p.Trp94X | n/a | n/a | Operating multiple keys with support |
| P14 (m) | *PAFAH1B1* | Whole gene deletion incl. *METTL16*, *PAFAH1B1* and *KIAA0664*; *YWHAE* not affected | n/a | 3-1, GPA, LAP | Directed arm movement |
| P15 (m) | *PAFAH1B1* | c.281G>A; p.W94* (Mosaic, 18% mutant allele) | n/a | n/a | Walking with support |
| P16 (f) | *PAFAH1B1* | Deletion with haplo insufficiency (*YWHAE* not affected) | n/a | n/a | Rolling over |
| P17 (f) | *PAFAH1B1* | Microdeletion 17p13.3 (*YWHAE* not affected) | n/a | 3-1, GPA, LAP | Rolling over |
| P18 (m) | *PAFAH1B1* | Deletion starting from exon 8 | n/a | n/a | Head control |
| P19 (m) | *PAFAH1B1* | c.162delA | n/a | 3-1, GPA, LAP | Operating a single key |
| P20 (m) | *PAFAH1B1* | Deletion exon 6-11 (210kb) | n/a | n/a | Head control |
| P21 (m) | *PAFAH1B1* | *PAFAH1B1* duplication (intron 1-2; 18kb) | De novo | 3-1, GPA, LAP | Free walking |
| P22 (m) | *PAFAH1B1* | c.22delC; p.R8Efs*4 | n/a | 3-1, GPA, LAP | Rolling over |
| P23 (f) | *PAFAH1B1* | Deletion exon 2-11 | n/a | n/a | Operating a single key with support |
| P24 (f) | *PAFAH1B1* | c.817C>T; p.Arg273X | n/a | 3-2, GPA, LDA | Holding onto an object single handed |
| P25 (f) | *PAFAH1B1* | c.749C>A; p.Ala250Asp | De novo | n/a | Free walking |
| P26 (m) | *PAFAH1B1* | c.656G>A; p.Trp219X | n/a | n/a | Operating a single key |
| P27 (f) | *PAFAH1B1* | c.1050delG; p.Lys351Ser*fs**4 | De novo | n/a | Head control |
| P28 (m) | *PAFAH1B1* | Complete *PAFAH1B1* deletion (*YWHAE* not affected) | n/a | 3-1, GPA, LAP | Rolling over |
| P29 (m) | *PAFAH1B1* | Complete *PAFAH1B1* deletion (*YWHAE* not affected) | n/a | 3-1, GPA, LAP | Simple arm movement |
| P30 (m) | *PAFAH1B1* | c.380delC; p.Ser127* | De novo | 3-1, GPA, LAP | Crawling |
| P31 (f) | *PAFAH1B1^+^* | *PAFAH1B1*-deletion (incl. *YWHAE*, *HIC1*, *METTL16*, *KIAA0664*) | De novo | 3-2, GD, LDA | Holding onto an object |
| P32 (f) | *PAFAH1B1* | Partial *PAFAH1B1* deletion 5‘ UTR exon 1 | De novo | n/a | Rolling over |
| P33 (m) | *PAFAH1B1* | Deletion exon 1 | n/a | n/a | Rolling over |
| P34 (m) | *PAFAH1B1* | c.162dupA; p.Trp55fs | De novo | n/a | Crawling |
| P35 (f) | *PAFAH1B1* | c.163T>C; p.Trp55Arg | De novo | 3-1, GPA, LAP | Walking with support |
| P36 (f) | *PAFAH1B1* | c.337C>T; .Arg113* | De novo | 3-1, GPA, LAP | Free sitting |
| P37 (m) | *PAFAH1B1* | Deletion 17p13.3p13.2 (1.99Mb;*YWHAE* not affected) | De novo | 3-1, GPA, LAP | None |
| P38 (m) | *PAFAH1B1^+^* | Microdeletion 17p13.2-p13.3 incl. *YWHAE* | De novo | 3-3, GD, LDA | None |
| P39 (m) | *DCX* | p.Arg192Trp (p.R192W) | Maternal | 3-1, GAP, LAP | Simple arm movement |
| P40 (m) | *DCX* | c.532C>T; p.Arg178Cys | Maternal | 1-3, GPA, LPP | Free walking |
| P41 (m) | *DCX* | c.586C>A ; p.Arg196Ser (p.R196S) | Maternal | 2-3, GAP, LDP | Free walking |
| P42 (m) | *DCX* | c.574C>T; p.Arg192Trp (p.R192W) | De novo | 3-1, GFP, LAP | Free sitting |
| P43 (m) | *DCX* | c.632T>C; p.Leu211Pro (p.L211P) | n/a | 3-2, GD, LDA | Crawling |
| P44 (m) | *DYNC1H1* | c.1103G>A; p.Arg368Gln | De novo | 2-3, GPA, LDP | Crawling |
| P45 (f) | *DYNC1H1* | c.6880G>A; p.Glu2294Lys | De novo | 1-3, GAP, LPP | Free walking |
| P46 (f) | *TUBA1A* | c.1204C>T; p.Arg402Cys | De novo | n/a | Rolling over |
| P47 (m) | *TUBG1* | c.776C>T; p.Ser259Leu | De novo | 1-3, GPA, LPP | Walking with support |
